# Efficacy and safety of semaglutide injection in comparison with reference semaglutide for chronic weight management in indian adults with obesity: A phase III randomized non-inferiority trial

**DOI:** 10.1016/j.metop.2026.100460

**Published:** 2026-03-23

**Authors:** Prabhat Kumar Sharma, Sagar Vivek Redkar, Abhishek Madhav Karmalkar, Sumit Anand, Vijaykumar Shivajirao Patil, Ansari Rizwanahmed Nurulhasan, Patel Manan Bharatkumar, Vijaykumar Barge, Shalin J. Shah, Chintan B. Patel, Animesh Choudhary, Kanugula Sudheer, Konatham Rambabu, S. Narasinga Rao, M. Swapna, Shravan Kumar Ankathi, Veer Bahadur Singh, Gouranga Sarkar, S.K. Gautam, Kalyan Kumar Das, Raja Bhattacharya, Kaushalendra Nath Tripathi, Budithi Sudarsi, Deven Parmar, Kevin Kansagra, Hardik Pathak

**Affiliations:** a Department of Medicine, Maharaja Agrasen Superspeciality Hospital, Central Spine, Agrasen Aspatal Marg, Sector 7, Vidyadhar Nagar, Jaipur, 302039, Rajasthan, Jaipur, 302039, Rajasthan, India; b Department of Medicine, Redkar Hospital and Research Centre, Mumbai-Goa Highway, Oxelbag, Dhargal, Tal-Pernem, Goa, 403513, North Goa, 403513, Goa, India; c Department of Medicine, Vedant Multispeciality Hospital, GP-83, Opp. to Rotary Club, Sambhaji Nagar, MIDC, Chinchwad, Pune, 411019, Maharashtra, Pune, 411019, Maharashtra, India; d Department of Medicine, NIMS Heart and Brain Hospital, B28, 29, Govind Marg, Raja Park, Jaipur, 302004, Rajasthan, Jaipur, 302004, Rajasthan, India; e Department of Medicine, Prakash Institute of Medical Sciences & Research (PIMS&R), Urun-Islampur, Islampur-Sangali Road, Islampur, Tal-Walwa, Dist-Sangali, 415409, Maharashtra, Sangali, 415409, Maharashtra, India; f Department of Medicine, Hope Well Medical Hospital, Block G, 1st Floor, 101, 102, Sumel-8, Nr. Ajit Mill Char Rasta, Rakhiyal, Ahmedabad, 380023, Gujarat, Ahmedabad, 380023, Gujarat, India; g Department of Medicine, Ananta Multispeciality Hospital, 4th Floor (416-418) Centre Point, Opp. Vrundavan Heights, Vande Mataram City to Savvy Swaraj Road, Chenpur, Gota, Ahmedabad, 382470, Gujarat, Ahmedabad, 382470, Gujarat, India; h Department of Medicine, Rajarshee Chhatrapati Shahu Maharaj Govt. Medical College and Chhatrapati Pramila Raje General Hospital, Dasara Chowk, Town Hall, Bhausingji Road, Kolhapur, 416012, Maharashtra, Kolhapur, 416012, Maharashtra, India; i Department of Medicine, Sheth Vadilal Sarabhai General Hospital & Sheth Chinai Maternity Hospital, Madalpur Gam, Paldi Road, Ellisbridge, Paldi, Ahmedabad, 380006, Gujarat, Ahmedabad, 380006, Gujarat, India; j Department of Medicine, Aatman Hospital, 5, Anveshan Row House, Opp. Umiya Mata Mandir, Bopal-Ghuma Main Road, Bopal, Ahmedabad, 380058, Gujarat, India; k Department of Medicine, SMC Heart Institute and IVF Research Centre, Infront of BSNL Office, Vidhan Sabha Road, Khamardih, Raipur, 492007, Chhattisgarh, Raipur, 492007, Chhattisgarh, India; l Department of General Medicine, Great Eastern Medical School and Hospital, Ragolu, Srikakulam, 532484, Andhra Pradesh, Srikakulam, 532484, Andhra Pradesh, India; m Department of General Medicine, Visakha Institute of Medical Sciences, NH-16, Hanumanthavaka Junction, Visakhapatnam, 530040, Andhra Pradesh, Visakhapatnam, 530040, Andhra Pradesh, India; n Department of Medicine, Rajendra Prasad Ward, Maharanipeta, Visakhapatnam, 530002, Andhra Pradesh, Visakhapatnam, 530040, Andhra Pradesh, India; o Department of General Medicine, In Patient Block, 3rd Floor, Gandhi Hospital, Musheerabad, Secunderabad, 500003, Telangana, Secunderabad, 500003, Telangana, India; p Department of Endocrinology, Mahatma Gandhi Memorial Hospital, Sherpura, Warangal, 506002, Telangana, Warangal, 506002, Telangana, India; q Department of Medicine, Sardar Patel Medical College & A.G. Hospitals, Sardar Patel Colony, Bikaner, 334001, Rajasthan, Bikaner, 334001, Rajasthan, India; r Department of General Medicine, Life Line Diagnostic Centre Cum Nursing Home, 4A, Wood Street, Kolkata, 700016, West Bengal, Kolkata, 700016, West Bengal, India; s Post Graduate Department of Medicine, LLR Hospital, GSVM Medical, College, Swaroop Nagar, Kanpur, 208002, Uttar Pradesh, Kanpur, 208002, Uttar Pradesh, India; t Department of Medicine, Aastha Hospital, Beside NBMCH, Opp. Bharat Petrol Pump, Kawakhari, Siliguri, 734012, West Bengal, Siliguri, 734012, West Bengal, India; u Department of Medicine, Medical College and Hospital, Kolkata, MCH Building, 4th Floor, 88 College Street, Kolkata, 700073, West Bengal, India; v Department of Medicine, Motilal Nehru Medical College & Associate Swarooprani Nehru Hospital, Prayagraj, 211001, Uttar Pradesh, Prayagraj, 211001, Uttar Pradesh, India; w Department of General Medicine, Government General Hospital, Cantonment, Vizianagaram, 535003, Andhra Pradesh, India; x Clinical R & D, Zydus Research Centre, Sarkhej-Bavla National Highway No. 8A Moraiya, Ahmedabad, 382213, Gujarat, India, Ahmedabad, 382213, Gujarat, India

**Keywords:** Semaglutide, Obesity, GLP-1 receptor agonist, Weight loss, Non-inferiority, India

## Abstract

**Background:**

In India, approximately 33-46% people are obese which is a major risk factor to several non-communicable diseases. Amongst all existing treatment modalities, semaglutide injection is the proven most effective glucagon-like peptide-1 (GLP-1) receptor agonist for obesity, but high costs limit global accessibility. We report the Phase III trial evaluating the efficacy, safety and immunogenicity of a novel formulation of Semaglutide Injection in Indian patients with obesity.

**Methods:**

This multicentre, randomized, open-label, active-controlled trial enrolled 282 Indian adults with obesity (BMI ≥30 kg/m^2^) or overweight with comorbidities across geographically distributed 23 sites. Participants were randomized 2:1 to Semaglutide Injection 15 mg/3 mL (5 mg/mL) (Test Product; Zydus Lifesciences Ltd.) or Reference Semaglutide (0.25 mg, 0.5 mg, 1 mg, 1.7 mg, and 2.4 mg) prefilled pen (Comparator Product; Novo Nordisk) for 24 weeks. Both arms followed an identical dose-escalation schedule from 0.25 mg to 2.4 mg once weekly. The primary endpoint was percentage weight change, with a non-inferiority margin of 5 percentage points.

**Results:**

In terms of primary endpoint, Least squares mean (LSM) was −11.25% (Test Product) versus −11.50% (reference semaglutide), with a treatment difference of 0.25% (95% CI: −0.72 to 1.23), at the end of 24 weeks confirming non-inferiority. 90.4% and 58.0% of Test Product-treated participants achieved ≥5% and ≥10% weight loss, respectively. Adverse events were similar between groups (63.3% vs 62.8%), with predominantly mild gastrointestinal symptoms. No serious treatment-related adverse events, deaths, or discontinuations occurred.

**Conclusions:**

This novel formulation of Semaglutide Injection demonstrated therapeutic non-inferiority to the reference product with comparable safety and immunogenicity.

**Trial registration:**

Clinical Trials Registry–India (CTRI/2025/03/082620)

## Highlights

• A Phase III randomized non-inferiority trial compared a novel semaglutide injection with the reference biologic in Indian adults with obesity.
• The test semaglutide demonstrated non-inferior weight loss to the reference product (−11.25% vs −11.50%) at 24 weeks.
• Over 90% of participants receiving the test product achieved clinically meaningful ≥5% weight loss.
• Safety and tolerability profiles were comparable between treatments, with predominantly mild gastrointestinal adverse events.
• No treatment-induced immunogenicity or serious treatment-related adverse events were observed.

## 1 Introduction

Obesity is a multifactorial, chronic and relapsing disease characterized by excess adiposity that adversely affects health. It is a significant risk of type 2 diabetes mellitus, cardiovascular disease, dyslipidemia, hypertension, musculoskeletal disorders, and certain cancers [1]. The prevalence of obesity has risen alarmingly, with every 1 in 8 people classified as obese, accounting for nearly 13% of the world's adult population (World Health Organisation, 2022) [2]. In India, the burden of obesity has increased at an alarming rate over the past two decades with prevalence estimates ranging from 33 to 46 percent depending on the region and the criteria applied [3]. Research results published by the National Family Health Survey demonstrate that obesity and overweight have grown in both genders, with men and women becoming more prevalent over the years since the early 2000s [4].

The Indian populations specifically show a prevalence of central obesity even among individuals with a relatively low body mass index indicating a higher proportion of visceral adiposity [5]. Despite the growing burden, the existing management strategies remain inadequate. Lifestyle modification with dietary changes, physical activity and behavioural interventions is the cornerstone therapy but is associated with modest weight reduction and frequent weight regain [6]. Orlistat, naltrexone/bupropion, phentermine/topiramate, and liraglutide are pharmacological interventions with demonstrated effectiveness in causing weight loss, but limited utility due to small effect sizes, gastrointestinal adverse effects, intolerability, and adherence problems [7].

Bariatric surgery achieves significant and lasting weight loss but is associated with high invasiveness, resource consumption, and is not feasible for the majority in low- and middle-income countries [8]. This poses a major gap in therapeutic options and therefore there is a necessity to develop effective and sustainable pharmacological agents. The glucagon-like peptide-1 (GLP-1) receptor agonists have emerged as an entirely novel class of drug for the treatment of obesity. Semaglutide, a long-acting GLP-1 receptor agonist administered once weekly, has demonstrated unprecedented efficacy in the Semaglutide Treatment Effect in People with obesity (STEP) clinical trial program. Among individuals without diabetes, a mean weight reduction of 14.9% to 17.4% was achieved over 68 weeks, accompanied by significant improvements in cardiometabolic risk factors [9].

Longer-term data has confirmed that semaglutide maintains weight loss over two years and cardiovascular outcome trials have further shown the benefit of semaglutide in mitigating adverse cardiac events [10]. The most common adverse events are gastrointestinal, mostly of mild-to-moderate severity and are manageable [11]. Given the superior efficacy of semaglutide as compared with previously available agents, semaglutide represents a major advancement in the management of obesity. However, its widespread use is hindered by cost and accessibility constraints, particularly in resource-limited settings such as India. Biosimilar development provides the opportunity to increase access to this therapy at a reduced cost, while ensuring similar efficacy and safety [12].

The weight-reducing effects of semaglutide are mediated through GLP-1 receptor activation in central nervous system appetite regulatory centres, leading to reduced hunger, enhanced satiety, and decreased food intake [13]. Additionally, GLP-1 receptor agonists delay gastric emptying, contributing to prolonged postprandial fullness. Body composition analyses in prior semaglutide trials have demonstrated that approximately 75% of weight loss represents fat mass, with preservation of lean mass, a ratio favourable compared to weight loss achieved through caloric restriction alone [14].

## 2 Methods

### 2.1 Study design and ethical considerations

This multicenter, randomized, active-controlled, open-label, parallel-group, Phase 3 clinical trial was conducted across geographically distributed 23 sites in India to evaluate the efficacy and safety of semaglutide injection in patients with obesity. Of 313 screened participants, 282 participants met the eligibility criteria and were randomized in a 2:1 ratio to receive either the Test product (semaglutide injection) or the reference semaglutide over a treatment period of 24 weeks. The protocol was reviewed and approved by the Drug Controller General of India (DCGI) and permission to conduct the present trial was granted by Central Drugs Standard Control Organization (CDSCO) on February 28, 2025. Ethics committee approval was obtained from institutional/independent ethics committees at all the respective participating centres.

### 2.2 Ethical considerations

The trial was prospectively registered with the Clinical Trials Registry–India (CTRI/2025/03/082620). Written informed consent to participate was obtained from all patients prior to any study-specific procedures. The study was conducted in accordance with the ethical principles of the Declaration of Helsinki, the International Council for Harmonisation–Good Clinical Practice (ICH) and all applicable local regulatory requirements.

### 2.3 Participants

Adults aged 18–75 years with a BMI ≥30 kg/m^2^, or ≥27 kg/m^2^ with at least one comorbidity (hypertension, dyslipidemia, or type 2 diabetes), were eligible for enrolment. Inclusion criteria required a history of at least one self-reported unsuccessful dietary effort to lose body weight, willingness to adhere to lifestyle interventions, and the ability to provide written informed consent.

The exclusion criteria were as follows: (a) General criteria applicable to all participants, prior treatment with any GLP-1 receptor agonist within 180 days or prior semaglutide use at any time, use of obesity pharmacotherapy within 90 days, self-reported body weight change >5 kg within 90 days, prior or planned bariatric surgery, uncontrolled thyroid disease (TSH >6.0 or <0.4 mIU/L), history of major depressive disorder within 2 years, severe psychiatric disorders, suicidal ideation or behaviour, history of pancreatitis, calcitonin ≥50 ng/L, personal or family history of MEN type 2 or medullary thyroid carcinoma, severe renal impairment (eGFR <30 mL/min/1.73 m^2^), elevated hepatic enzymes (AST, ALT, ALP, or total bilirubin >1.5 × ULN), malignancy within 5 years, recent cardiovascular events (myocardial infarction, stroke, or unstable angina within 60 days), heart failure (NYHA Class II–IV), known hypersensitivity to study products, and pregnancy or breastfeeding. (b) Specific criteria for non-diabetic participants: HbA1c ≥ 6.5% or use of glucose-lowering agents within 90 days. (c) Specific criteria for diabetic participants: use of glucose-lowering agents other than the permitted oral agents (metformin, sulfonylureas, SGLT2 inhibitors, or thiazolidinediones), or presence of uncontrolled diabetic retinopathy or maculopathy.

### 2.4 Randomization and masking

Randomization was performed centrally with a computer-generated sequence in a 2:1 ratio to assign the participants to the test semaglutide injection (Test Product) or the reference semaglutide. The Test product (15mg/3 ml[5 mg/ml], produced by Zydus Lifesciences Ltd.) was administered using a single multi-dose pen containing a total of 15 mg semaglutide in a single cartridge, designed to deliver all protocol-specified dose levels (0.25 mg, 0.5 mg, 1 mg, 1.7 mg, and 2.4 mg). Each cartridge was sufficient for approximately 8 weeks of dosing, and new cartridges were dispensed at Weeks 0, 8, and 16. The reference semaglutide consisted of prefilled pens at corresponding dose strengths (0.25 mg, 0.5 mg, 1 mg, 1.7 mg, and 2.4 mg), manufactured by Novo Nordisk. Given differences in product presentations, the trial was conducted in an open-label manner; however, efficacy data were analysed by independent statisticians who were blinded to treatment allocation.

### 2.5 Procedures

After a screening period of up to two weeks, eligible patients were randomized at baseline (Week 0). Study drugs were administered once weekly by subcutaneous injection (abdomen, thigh, or upper arm) at any time of day, regardless of meals. Both treatment arms followed an identical fixed dose-escalation schedule to improve tolerability: 0.25 mg once weekly (Weeks 0–4), 0.5 mg (Weeks 4–8), 1.0 mg (Weeks 8–12), 1.7 mg (Weeks 12–16), and 2.4 mg (Weeks 16–24, maintenance). Patients unable to tolerate the 2.4 mg maintenance dose were permitted to down-titrate to 1.7 mg once weekly. All participants received standardized diet and exercise counselling (minimum 150 min of physical activity per week), maintained adherence diaries, and if patients were diabetic, glucometers were used for self-monitoring. Diabetic patients using sulfonylureas reduced their sulfonylurea dose by approximately 50% at randomization to minimize hypoglycaemia risk. Study visits occurred at Weeks 4, 8, 12, 16, 20, and 24, with a telephonic safety follow-up at Week 28.

### 2.6 Endpoints

The primary endpoint was the percentage change in body weight from baseline to Week 24. Secondary endpoints included interim weight changes (Weeks 4 to 20), proportions achieving ≥5% and ≥10% weight loss, and changes in BMI, waist circumference, and SF-36 physical functioning scores. In type 2 diabetes patients, HbA1c was assessed at baseline, Week 12, and Week 24; fasting plasma glucose (FPG) and 2-h postprandial glucose (PPG) were assessed at baseline and all follow-up visits. In non-diabetic patients, HbA1c, FPG, and PPG were assessed at baseline and Week 24. Safety endpoints included adverse and serious adverse events, discontinuations due to adverse events, hypoglycemia (in diabetics), and anti-drug antibodies (ADA) and neutralizing antibodies (NAb) at baseline and Week 24.

### 2.7 Statistical analysis

A total of 282 patients (188 test, 94 reference; 2:1 ratio) were planned to provide 90% power to demonstrate non-inferiority, assuming α = 0.05, a 5% margin, SD ± 11, and ∼20% dropout. The primary efficacy analysis followed the intention-to-treat principle, including all randomized patients who received ≥1 dose; safety analyses included all treated participants. Continuous variables were summarized as means with standard deviations and 95% confidence intervals and were compared using ANOVA or mixed models, as appropriate. Categorical variables were analysed using chi-square or Fisher's exact tests. All statistical tests were two-sided (α = 0.05) and missing data were handled using the last observation carried forward (LOCF) method as prespecified in the statistical analysis plan.

## 3 Results

### 3.1 Patient disposition and baseline characteristics

**Fig. 1:**
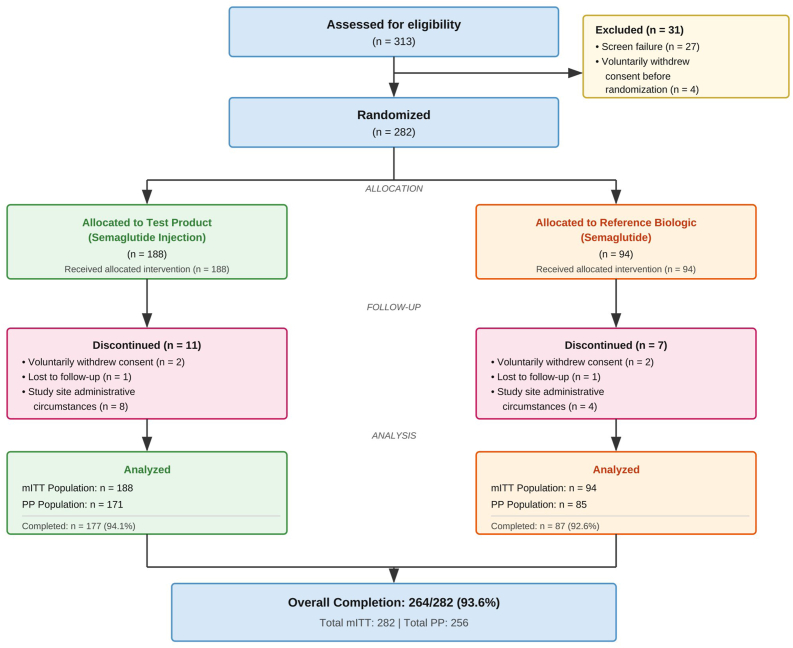
CONSORT Flow Diagram *mITT = modified intention-to-treat; PP = per-protocol. Test Product = Semaglutide Injection* 15 mg*/*3 mL *(*5 mg/mL*) - Zydus Lifesciences Ltd Reference Semaglutide = Reference Semaglutide Injection - Novo Nordisk.*

Between March and October 2025, 313 patients were screened across 23 study sites in India. Of these, 31 (9.9%) were not randomized: 27 failed eligibility criteria and 4 withdrew consent before randomization. A total of 282 patients met eligibility criteria and were randomized: 188 to the Test Product group and 94 to the reference semaglutide group. A total of 264 participants (93.6%) completed the 24-week treatment period, with 177 (94.1%) completers in the Test Product group and 87 (92.6%) in the reference semaglutide group. Eighteen participants discontinued prematurely: 4 voluntarily withdrew consent, 2 were lost to follow-up, and 12 were withdrawn due to investigator or CRO owing to administrative circumstances such as site-specific study termination. No discontinuations were attributed to adverse events. The mITT population comprised all 282 randomized participants, and the PP population included 256 participants (171 Test, 85 reference) who completed the study without major protocol deviations as illustrated in Fig. 1. Assessment completeness at Week 24 was 94.1% (177/188) in the Test Product group and 92.6% (87/94) in the reference semaglutide group. Missing efficacy data for participants who discontinued prematurely were imputed using the last observation carried forward (LOCF) method. No efficacy data were missing for participants who completed the study.

As seen in Table 1, the baseline demographic and clinical characteristics were well balanced between treatment groups. All participants were of Asian Indian ethnicity.

**Table 1:** Baseline demographic and clinical characteristics.

Characteristic	Test Product (N = 188)	Reference Semaglutide (N = 94)	P-value
Age, years, mean (SD)	44.93 (12.82)	43.59 (11.31)	0.3904
Median (Min, Max)	43.50 (18.00, 75.00)	43.00 (21.00, 67.00)	
Sex, n (%)			0.6338
Female	96 (51.1%)	41 (43.6%)	
Male	92 (48.9%)	53 (56.4%)	
Body weight, kg, mean (SD)	86.75 (13.22)	88.62 (16.57)	0.3068
Median (Min, Max)	84.75 (65.00, 144.60)	86.75 (64.60, 179.00)	
BMI, kg/m^2^, mean (SD)	33.85 (5.39)	33.78 (5.36)	0.9155
Median (Min, Max)	32.23 (27.34, 55.03)	32.36 (27.15, 54.01)	
Height, cm, mean (SD)	160.30 (7.23)	161.88 (8.25)	0.1011
Waist circumference, cm, mean (SD)	101.97 (15.04)	101.00 (14.24)	0.6024
Comorbidities, n (%)			
Hypertension	66 (35.1%)	30 (31.9%)	
Type 2 diabetes mellitus	54 (28.7%)	28 (29.8%)	
Dyslipidemia	15 (8.0%)	7 (7.4%)	
Hypothyroidism	6 (3.2%)	5 (5.3%)	
Obstructive sleep apnea	1 (0.5%)	0 (0.0%)	
Ethnicity: Asian Indian, n (%)	188 (100.0%)	94 (100.0%)	

Abbreviations: BMI, body mass index; mITT, modified intention-to-treat; SD, standard deviation. P-values from two-sample *t*-test (continuous variables) or chi-square test (categorical variables). All p-values >0.05, confirming adequate balance between groups.

### 3.2 Primary endpoint

**Fig. 2:**
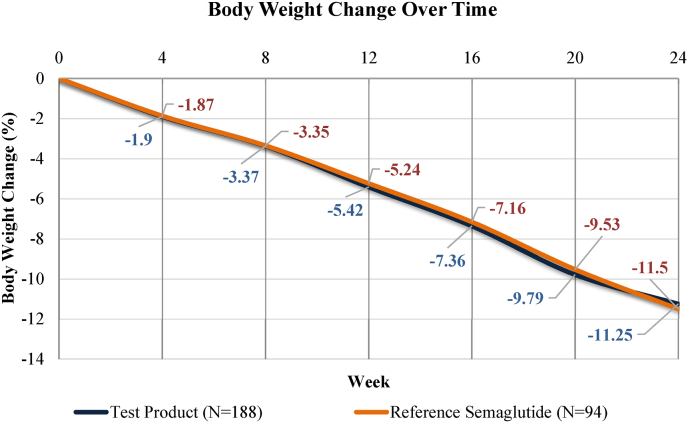
Percentage change in body weight from baseline to week 24. Both groups demonstrated progressive and comparable weight loss throughout the 24-week treatment period, with mean weight reductions of −11.25% (Test Product) and −11.5% (Reference Semaglutide) at week 24.

The primary endpoint of percentage change in body weight from baseline to Week 24 was achieved, demonstrating non-inferiority of Test Product to reference semaglutide as seen in Table 2. In the mITT population, the LSM percentage change in body weight was −11.25% (SE 0.29) in the Test group and −11.50% (SE 0.41) in the reference group. The LSM difference was 0.25% (95% CI: −0.72 to 1.23; P = 0.613), with the upper bound of the 95% CI (1.23%) well below the pre-specified non-inferiority margin of 5 percentage points (Fig. 2).

**Table 2:** Primary and secondary efficacy outcomes: Body weight (mITT population).

Outcome	Test Product(N = 188)	Reference (N = 94)	LSM Difference (95% CI)	P-value
**Primary Endpoint**				
% weight change, Week 24, LSM (SE)	−11.25 (0.29)	−11.50 (0.41)	0.25 (−0.72, 1.23)	0.613
**Secondary Endpoints: % Weight Change Over Time, LSM (SE)**				
Week 4	−1.90 (0.30)	−1.87 (0.43)	−0.04 (−1.07, 0.99)	0.946
Week 8	−3.37 (0.29)	−3.35 (0.41)	−0.02 (−1.00, 0.96)	0.969
Week 12	−5.42 (0.29)	−5.24 (0.41)	−0.18 (−1.16, 0.80)	0.720
Week 16	−7.36 (0.29)	−7.16 (0.41)	−0.20 (−1.17, 0.78)	0.689
Week 20	−9.79 (0.29)	−9.53 (0.41)	−0.26 (−1.24, 0.71)	0.599
**Responder Analysis at Week 24, n (%)**				
≥5% weight loss	170 (90.4%)	83 (88.3%)	—	0.540
≥10% weight loss	109 (58.0%)	54 (57.4%)	—	0.351

Abbreviations: CI, confidence interval; LSM, least squares mean; mITT, modified intention-to-treat; SE, standard error. LSM and P-values from repeated measures ANCOVA with baseline body weight as covariate. Non-inferiority margin: 5 percentage points.

Results in the PP population were consistent with the primary analysis. The LSM percentage change was −11.78% (SE 0.29) in the Test group and −12.11% (SE 0.41) in the reference group, with an LSM difference of 0.33% (95% CI: −0.66 to 1.32; P = 0.513), confirming non-inferiority (Supplementary Table S1).

### 3.3 Secondary efficacy endpoints

*Weight change over time:* Progressive weight loss was observed throughout the 24-week treatment period in both groups, with no significant between-group differences at any time point (Table 2). At Week 4, LSM weight change was −1.90% versus −1.87% (P = 0.946); at Week 12, −5.42% versus −5.24% (P = 0.720); and at Week 20, −9.79% versus −9.53% (P = 0.599) for Test and reference groups, respectively.

*Responder analysis:* At Week 24, the proportion of participants achieving ≥5% weight loss was 90.4% (170/188) in the Test group versus 88.3% (83/94) in the reference group (P = 0.540). The proportion achieving ≥10% weight loss was 58.0% (109/188) versus 57.4% (54/94), respectively (P = 0.351). No significant differences were observed between groups at any time point (Table 2).

*Dosing and adherence:* All participants who completed the study (177 Test Product, 87 reference semaglutide) achieved and maintained the target maintenance dose of 2.4 mg once weekly from Week 16 onwards. No dose reductions to 1.7 mg were required in either treatment group. Mean study participation was 187.23 days (Test Product) and 184.32 days (reference semaglutide). Dose-by-visit data are presented in Supplementary Table S4.

*Anthropometric outcomes:* As shown in Table 3, both groups demonstrated substantial improvements in anthropometric parameters. BMI decreased by a mean of 3.79 kg/m^2^ (Test) versus 3.87 kg/m^2^ (reference) at Week 24 (LSM difference 0.08; 95% CI: −0.26 to 0.42; P = 0.648). Waist circumference decreased by 10.54 cm versus 10.19 cm (LSM difference −0.35; 95% CI: −1.71 to 1.01; P = 0.615). SF-36 Physical Functioning scores improved by 24.3 points versus 23.1 points (P = 0.608).

**Table 3:** Changes in anthropometric and metabolic parameters from baseline to week 24 (mITT population).

Parameter	Test Product	Reference	Difference (95% CI)	P-value
**All Patients**				
BMI change, kg/m^2^, LSM (SE)	−3.80 (0.10)	−3.88 (0.14)	0.08 (−0.26, 0.42)	0.648
Waist circumference change, cm, LSM (SE)	−10.54 (0.40)	−10.19 (0.57)	−0.35 (−1.71, 1.01)	0.615
SF-36 Physical Functioning change, mean (SD)	+24.3 (18.2)	+23.1 (17.5)	—	0.608
**Patients with Type 2 Diabetes (n=82)**				
HbA1c change, %, LSM (SE)	−1.52 (0.12)	−1.80 (0.17)	0.27 (−0.14, 0.68)	0.195
Baseline HbA1c, %, mean (SD)	8.32 (0.71)	8.30 (0.87)	—	—
Week 24 HbA1c, %, mean (SD)	6.80 (0.77)	6.56 (0.81)	—	—
FPG change, mg/dL, LSM (SE)	−42.09 (4.51)	−49.07 (6.26)	6.98 (−8.18, 22.13)	0.366
2-h PPG change, mg/dL, LSM (SE)	−62.84 (6.23)	−69.26 (8.65)	6.42 (−14.51, 27.35)	0.546
**Patients without Diabetes (n=200)**				
HbA1c change, %, LSM (SE)	−0.12 (0.06)	+0.03 (0.08)	−0.15 (−0.34, 0.05)	0.133
FPG change, mg/dL, LSM (SE)	−0.82 (1.73)	+3.97 (2.45)	−4.78 (−10.70, 1.14)	0.113

Abbreviations: BMI, body mass index; CI, confidence interval; FPG, fasting plasma glucose; HbA1c, glycated hemoglobin; LSM, least squares mean; PPG, postprandial glucose; SD, standard deviation; SE, standard error; SF-36, Short Form-36 Health Survey.

*Glycemic outcomes in diabetic subgroup:* Among participants with type 2 diabetes (n = 82), both groups showed clinically meaningful improvements in glycemic control. HbA1c decreased from a baseline of 8.32% (SD 0.71) to 6.80% (SD 0.77) in the Test group and from 8.30% (SD 0.87) to 6.56% (SD 0.81) in the reference group. The LSM change was −1.52% versus −1.80% (LSM difference 0.27; 95% CI: −0.14 to 0.68; P = 0.195). Fasting plasma glucose decreased by 42.1 mg/dL versus 49.1 mg/dL (P = 0.366), and 2-h postprandial glucose decreased by 62.8 mg/dL versus 69.3 mg/dL (P = 0.546). No significant between-group differences were observed for any glycemic parameter (Table 3). Comparative results for per protocol analysis are presented in Supplementary Table S2.

### 3.4 Safety

A total of 458 adverse events were reported in 178 participants (63.1%) during the study as can be seen in Table 4. The incidence of any adverse event was similar between groups: 63.3% (119/188) in the Test Product group and 62.8% (59/94) in the reference semaglutide group (P = 0.926, chi-square test). No deaths, serious adverse events, or discontinuations due to adverse events occurred in either group.

**Table 4:** Summary of adverse events (safety population).

Category	Test Product (N = 188)	Reference Semaglutide (N = 94)
**Overview**		
Patients with any AE, n (%)	119 (63.3%)	59 (62.8%)
Total number of AEs	318	140
Serious adverse events	0 (0%)	0 (0%)
Deaths	0 (0%)	0 (0%)
Discontinuation due to AE	0 (0%)	0 (0%)
**Severity of AEs (number of events)**		
Mild	308	138
Moderate	10	2
Severe	0	0
**AEs by System Organ Class (number of events)**		
**Gastrointestinal disorders**	256	117
Diarrhoea	70	20
Vomiting	64	21
Nausea	47	30
Bloating	25	14
Acidity	17	7
Dyspepsia	10	5
Constipation	7	14
Abdominal pain	6	2
Gastritis	2	3
Abdominal distension	2	1
Other GI events^a^	6	0
**General disorders and administration site conditions**	35	8
Pyrexia	19	3
Asthenia	14	4
Fatigue	2	0
Injection site pain	0	1
**Nervous system disorders**	10	8
Headache	8	5
Somnolence	2	0
Dizziness	0	2
Ageusia	0	1
**Metabolism and nutrition disorders**	8	2
Decreased appetite	8	2
**Musculoskeletal and connective tissue disorders**	4	1
Body pain	4	1
**Skin and subcutaneous tissue disorders**	3	2
Alopecia	3	0
Skin allergy	0	1
Pruritus	0	1
**Infections and infestations**	1	2
Nasopharyngitis	1	2
**Respiratory, thoracic and mediastinal disorders**	1	0
Cough	1	0

Adverse events coded using MedDRA. All AEs resolved without sequelae.

Note: Test Product = Semaglutide Injection 15 mg/3 mL (5 mg/mL) manufactured by Zydus Lifesciences Ltd. Reference Semaglutide = Reference Semaglutide Injection (Semaglutide) manufactured by Novo Nordisk.

a Other GI events include: epigastric discomfort (1), flatulence (2), gastrointestinal disorder (1), heartburn (1), toothache (1) in Test Product group.

The majority of adverse events were mild in severity (446/458, 97.4%), with 12 moderate events (10 in the Test Product group and 2 in the reference semaglutide group; 2.6%) and no severe events. Gastrointestinal disorders were the most frequently reported, accounting for 373 of 458 total events (81.4%), consistent with the known pharmacology of GLP-1 receptor agonists. The most common adverse events were diarrhoea (19.7%), vomiting (18.6%), nausea (16.8%), bloating (8.5%), and acidity (5.2%). General disorders (primarily pyrexia and asthenia) accounted for 9.4% of events. All adverse events resolved without sequelae.

Notably, no cases of acute pancreatitis, gastroparesis, intestinal obstruction, gallbladder-related events, or cardiac adverse events were reported in either treatment group. Injection site pain was reported in 2 participants (1.06%) in the Test Product group and none in the reference semaglutide group. Adverse events by system organ class are presented in Table 5, and a detailed listing by preferred term is provided in Supplementary Table S5.

**Table 5:** Adverse events by system organ class (safety population).

System Organ Class	Test Product (N = 188)Events	Reference Semaglutide (N = 94)Events	Total (N = 282)Events
Gastrointestinal disorders	256	117	373
General disorders and administration site conditions	35	8	43
Nervous system disorders	10	8	18
Metabolism and nutrition disorders	8	2	10
Musculoskeletal and connective tissue disorders	4	1	5
Skin and subcutaneous tissue disorders	3	2	5
Infections and infestations	1	2	3
Respiratory, thoracic and mediastinal disorders	1	0	1
**Total**	**318**	**140**	**458**

Adverse events coded using MedDRA. All AEs resolved without sequelae.

Laboratory assessments including hematological parameters (hemoglobin, hematocrit, RBC count, platelet count, WBC count with differential), biochemical parameters (total bilirubin, AST, ALT, ALP, BUN, serum creatinine, eGFR, serum uric acid, serum calcitonin, amylase, and lipase), lipid profile (triglycerides, total cholesterol, LDL, HDL), and routine urinalysis were performed at baseline and Week 24. No clinically significant changes were observed in any laboratory parameters in either treatment group. Vital signs including pulse rate, respiratory rate, body temperature, and blood pressure (systolic and diastolic) were assessed at each study visit. Mean pulse rate showed a modest reduction from baseline to Week 24 in both groups (Test Product: −2.31 bpm; reference semaglutide: −1.99 bpm), while blood pressure remained stable with no clinically meaningful changes. Systemic examination findings, 12-lead electrocardiogram results, and retinal examination (fundoscopy) showed no clinically significant abnormalities throughout the study in either group.

### 3.5 Immunogenicity

Immunogenicity was assessed by measuring anti-drug antibodies (ADA) and neutralizing antibodies (NAb) at baseline (Day 0) and Week 24. Out of 282 randomized patients, 4 (1.42%) samples were ADA-positive at either pre-dose or post-dose assessment: 3 in the Test Product group (all at pre-dose) and 1 in the reference semaglutide group (at post-dose Week 24). Among the ADA-positive patients, 2 (0.71%) in the Test Product group tested positive for NAb at pre-dose. Importantly, no treatment-induced or treatment-boosted ADA or NAb responses were observed in either group, as all positive results were detected at baseline (pre-dose) timepoints, with the exception of one post-dose ADA-positive result in the reference semaglutide group. The overall incidence of ADA positivity was low and comparable between treatment arms, with no apparent impact on efficacy or safety outcomes (Supplementary Table S3).

## 4 Discussion

This Phase III randomized trial demonstrated non-inferiority of the Test Product (Semaglutide Injection 15 mg/3 mL) to reference semaglutide for chronic weight management in Indian adults with obesity. A distinguished feature of the Test Product lies in its multi-dose pen design utilizing a single 15 mg/3 mL (5 mg/mL) cartridge delivering all protocol-specified doses (0.25 mg, 0.5 mg, 1 mg, 1.7 mg, and 2.4 mg) during dose-escalation and maintenance periods, unlike the dose-specific separate pens for the reference product. New cartridges were administered at Weeks 0, 8, and 16. This single-device approach offers practical benefits including fewer storage requirements, reduced complexity in patient medication management, improved adherence, and decreased medical waste generation. Following the successful outcomes of the present clinical trial, Semaglutide Injection 15 mg/3 mL has received manufacturing and marketing approval from the DCGI office in January 2026.

The weight loss magnitude observed in our study aligns favourably with the results of the landmark STEP clinical trial program. In STEP 1, Wilding and colleagues reported a mean weight reduction of 14.9% at 68 weeks with semaglutide in adults without diabetes [15]. Our 24-week results of approximately 11% weight loss are consistent with the trajectory of weight loss observed at comparable timepoints in the STEP program. Similarly, in STEP 2, which enrolled adults with overweight or obesity and type 2 diabetes, semaglutide produced a mean weight loss of 9.6% at 68 weeks, with the lesser weight reduction compared to participants without diabetes [16]. Longer-duration studies are warranted to evaluate sustained efficacy beyond 24 weeks.

Our results are also consistent with the phase 2 dose-ranging trial by O'Neil and colleagues, which established the dose-response relationship for semaglutide in weight management [16]. In that 52-week study, semaglutide at doses equivalent to approximately 1.4 mg and 2.8 mg weekly produced mean weight losses of 11.2% and 13.8%, respectively, confirming that the 2.4 mg weekly maintenance dose used in our study represents an optimal balance between efficacy and tolerability.

The weight loss achieved in our study substantially exceeds that reported with other pharmacological interventions for obesity, including liraglutide 3.0 mg (8.0% at 56 weeks) [17], orlistat (5–6% beyond placebo) [18], phentermine-topiramate (8–10%) [19], and naltrexone-bupropion (approximately 5%) [20]. The efficacy of the Test Product at the 2.4 mg weekly maintenance dose thus positions it among the most effective currently available pharmacotherapies for obesity.

For assessing therapeutic response, categorical weight loss thresholds provide clinically meaningful benchmarks. In our study, 90.4% of Test Product-treated participants achieved clinically significant weight loss of ≥5%, and 58.0% achieved ≥10% weight loss at 24 weeks. These responder rates compare favourably with STEP 1, where 86.4% achieved ≥5% and 69.1% achieved ≥10% weight loss at 68 weeks. The similarity in categorical response rates despite our shorter treatment duration suggests comparable biological activity of the Test Product. Weight reductions of 5-10% are associated with clinically meaningful improvements in cardiometabolic risk factors, including reductions in blood pressure, glycemic parameters, and triglycerides, as well as improvements in health-related quality of life [21].

This study addresses an important unmet need in the Indian healthcare context. India faces a substantial burden of obesity, with the ICMR-INDIAB study reporting prevalence rates of generalized obesity (BMI ≥25 kg/m^2^) ranging from 11.8% to 31.3% across different states, and abdominal obesity affecting 16.9% to 39.5% of the adult population [22]. The 'Asian Indian phenotype,' characterized by higher body fat percentage and central adiposity at lower BMI values compared to Western populations, confers heightened susceptibility to obesity-related complications including type 2 diabetes and cardiovascular disease at lower BMI thresholds. Our study population, with a mean baseline BMI of 33.8 kg/m^2^, represents individuals at particularly high cardiometabolic risk. The availability of an effective and potentially more accessible Semaglutide Injection option may facilitate broader adoption of evidence-based obesity pharmacotherapy in India and similar low- and middle-income settings where the innovator product may be cost-prohibitive. However, evidence suggests that the pharmacodynamic response to semaglutide is preserved across ethnic groups despite differences in baseline metabolic phenotype [23]. This supports extrapolation of findings and use of semaglutide across diverse populations.

The safety profile of the Test Product was consistent with the known profile of GLP-1 receptor agonists. Gastrointestinal adverse events (nausea, vomiting, and diarrhoea) were mild to moderate in severity, transient in nature, and occurred most frequently during the dose-escalation period. In the STEP trials, gastrointestinal events led to treatment discontinuation in 4.5% of participants [23]. In our study, no patients discontinued treatment due to adverse events in either group, supporting the tolerability of semaglutide at all dose levels. No serious adverse events including acute pancreatitis, gallbladder disease, gastroparesis, intestinal obstruction, cardiac events, or severe hypoglycemia were reported.

The results of the study should be interpreted in the context of its limitations. First, the 24-week treatment duration is shorter than the 52–68 weeks duration of most obesity trials; longer-term studies are needed to confirm sustained efficacy and safety. Second, the open-label design may have introduced bias in subjective adverse event reporting, although the primary endpoint was objective and unlikely to be affected. Third, our study population was limited to Indian adults, and results may not be directly generalizable to other ethnic populations, although evidence suggests that the pharmacodynamic response to semaglutide is preserved across ethnic groups [24]. Fourth, systematic assessment of body composition was not performed, precluding analysis of fat versus lean mass changes.

## 5 Conclusions

This Phase III randomized trial demonstrated that Semaglutide Injection (Test Product) is non-inferior to reference semaglutide for chronic weight management in Indian adults with obesity. Both treatments achieved clinically meaningful weight loss of approximately 11% at 24 weeks, with comparable improvements in BMI, waist circumference, quality of life, and glycemic parameters in the diabetic subpopulation. The safety profile was similar between groups, characterized by predominantly mild-to-moderate gastrointestinal adverse events consistent with the GLP-1 receptor agonist class, and no serious treatment-related safety concerns were identified. No treatment-emergent immunogenic responses were observed in either treatment group. These findings support the Test Product as a therapeutically equivalent alternative to the reference product, potentially improving access to effective obesity pharmacotherapy in resource-constrained settings where the high cost of innovator biologics may limit treatment availability.

## CRediT authorship contribution statement

**Prabhat Kumar Sharma:** Writing – review & editing, Writing – original draft, Visualization, Validation, Supervision, Software, Resources, Project administration, Methodology, Investigation, Formal analysis, Data curation, Conceptualization. **Sagar Vivek Redkar:** Writing – review & editing, Writing – original draft, Visualization, Validation, Supervision, Software, Resources, Project administration, Methodology, Investigation, Formal analysis, Data curation, Conceptualization. **Abhishek Madhav Karmalkar:** Writing – review & editing, Writing – original draft, Visualization, Validation, Supervision, Software, Resources, Project administration, Methodology, Investigation, Formal analysis, Data curation, Conceptualization. **Sumit Anand:** Writing – review & editing, Writing – original draft, Visualization, Validation, Supervision, Software, Resources, Project administration, Methodology, Investigation, Formal analysis, Data curation, Conceptualization. **Vijaykumar Shivajirao Patil:** Writing – review & editing, Writing – original draft, Visualization, Validation, Supervision, Software, Resources, Project administration, Methodology, Investigation, Formal analysis, Data curation, Conceptualization. **Ansari Rizwanahmed Nurulhasan:** Writing – review & editing, Writing – original draft, Visualization, Validation, Supervision, Software, Resources, Project administration, Methodology, Investigation, Formal analysis, Data curation, Conceptualization. **Patel Manan Bharatkumar:** Writing – review & editing, Writing – original draft, Visualization, Validation, Supervision, Software, Resources, Project administration, Methodology, Investigation, Formal analysis, Data curation, Conceptualization. **Vijaykumar Barge:** Writing – review & editing, Writing – original draft, Visualization, Validation, Supervision, Software, Resources, Project administration, Methodology, Investigation, Formal analysis, Data curation, Conceptualization. **Shalin J. Shah:** Writing – review & editing, Writing – original draft, Visualization, Validation, Supervision, Software, Resources, Project administration, Methodology, Investigation, Formal analysis, Data curation, Conceptualization. **Chintan B. Patel:** Writing – review & editing, Writing – original draft, Visualization, Validation, Supervision, Software, Resources, Project administration, Methodology, Investigation, Formal analysis, Data curation, Conceptualization. **Animesh Choudhary:** Writing – review & editing, Writing – original draft, Visualization, Validation, Supervision, Software, Resources, Project administration, Methodology, Investigation, Formal analysis, Data curation, Conceptualization. **Kanugula Sudheer:** Writing – review & editing, Writing – original draft, Visualization, Validation, Supervision, Software, Resources, Project administration, Methodology, Investigation, Formal analysis, Data curation, Conceptualization. **Konatham Rambabu:** Writing – review & editing, Writing – original draft, Visualization, Validation, Supervision, Software, Resources, Project administration, Methodology, Investigation, Formal analysis, Data curation, Conceptualization. **S. Narasinga Rao:** Writing – review & editing, Writing – original draft, Visualization, Validation, Supervision, Software, Resources, Project administration, Methodology, Investigation, Formal analysis, Data curation, Conceptualization. **M. Swapna:** Writing – review & editing, Writing – original draft, Visualization, Validation, Supervision, Software, Resources, Project administration, Methodology, Investigation, Formal analysis, Data curation, Conceptualization. **Shravan Kumar Ankathi:** Writing – review & editing, Writing – original draft, Visualization, Validation, Supervision, Software, Resources, Project administration, Methodology, Investigation, Formal analysis, Data curation, Conceptualization. **Veer Bahadur Singh:** Writing – review & editing, Writing – original draft, Visualization, Validation, Supervision, Software, Resources, Project administration, Methodology, Investigation, Formal analysis, Data curation, Conceptualization. **Gouranga Sarkar:** Writing – review & editing, Writing – original draft, Visualization, Validation, Supervision, Software, Resources, Project administration, Methodology, Investigation, Formal analysis, Data curation, Conceptualization. **S.K. Gautam:** Writing – review & editing, Writing – original draft, Visualization, Validation, Supervision, Software, Resources, Project administration, Methodology, Investigation, Formal analysis, Data curation, Conceptualization. **Kalyan Kumar Das:** Writing – review & editing, Writing – original draft, Visualization, Validation, Supervision, Software, Resources, Project administration, Methodology, Investigation, Formal analysis, Data curation, Conceptualization. **Raja Bhattacharya:** Writing – review & editing, Writing – original draft, Visualization, Validation, Supervision, Software, Resources, Project administration, Methodology, Investigation, Formal analysis, Data curation, Conceptualization. **Kaushalendra Nath Tripathi:** Writing – review & editing, Writing – original draft, Visualization, Validation, Supervision, Software, Resources, Project administration, Methodology, Investigation, Formal analysis, Data curation, Conceptualization. **Budithi Sudarsi:** Writing – review & editing, Writing – original draft, Visualization, Validation, Supervision, Software, Resources, Project administration, Methodology, Investigation, Formal analysis, Data curation, Conceptualization. **Deven Parmar:** Writing – review & editing, Writing – original draft, Visualization, Validation, Supervision, Software, Resources, Project administration, Methodology, Investigation, Formal analysis, Data curation, Conceptualization. **Kevin Kansagra:** Writing – review & editing, Writing – original draft, Visualization, Validation, Supervision, Software, Resources, Project administration, Methodology, Investigation, Formal analysis, Data curation, Conceptualization. **Hardik Pathak:** Writing – review & editing, Writing – original draft, Visualization, Validation, Supervision, Software, Resources, Project administration, Methodology, Investigation, Formal analysis, Data curation, Conceptualization.

## Data statement

(State whether the data supporting the findings of this study are available in a publicly accessible repository (provide DOI/access link), or describe restrictions due to patient confidentiality, ethical, or commercial considerations)

The data supporting the findings of this study are not publicly available due to [patient confidentiality/contractual restrictions], but may be available from the corresponding author on reasonable request (subject to approval)

## Declaration of generative AI use

The authors declare that no generative AI tools were used in the writing of this manuscript.

## Funding statement

This study was funded by 10.13039/100032550Zydus Lifesciences.

## Declaration of competing interest

For authors no. 24, 25, 26: Dr Deven Parmar, Dr Kevinkumar Kansagra and Dr Hardik Pathak are employees of Zydus Lifesciences Ltd., Ahmedabad, India. All other authors declare no competing interests.

## References

[1] Chamarthi V.S., Daley S. (2025). Obesity.

[2] World Health Organization (2020). Obesity and overweight [Internet]. https://www.who.int/news-room/fact-sheets/detail/obesity-and-overweight.

[3] Venkatrao M., Nagarathna R., Majumdar V., Patil S.S., Rathi S., Nagendra H. (2020). Prevalence of obesity in India and its neurological implications: a multifactor analysis of a nationwide cross-sectional study. Ann Neurosci.

[4] Luhar S., Timaeus I.M., Jones R., Cunningham S., Patel S.A., Kinra S. (2020). Forecasting the prevalence of overweight and obesity in India to 2040. PLoS One.

[5] Misra A., Khurana L. (2011). Obesity-related non-communicable diseases: south Asians vs white Caucasians. Int J Obes.

[6] Bray G.A., Heisel W.E., Afshin A., Jensen M.D., Dietz W.H., Long M. (2018). The science of obesity management: an endocrine society scientific statement. Endocr Rev.

[7] Apovian C.M., Aronne L.J., Bessesen D.H., McDonnell M.E., Murad M.H., Pagotto U. (2015). Pharmacological management of obesity: an endocrine society clinical practice guideline. J Clin Endocrinol Metab.

[8] Maciejewski M.L., Arterburn D.E., Van Scoyoc L., Smith V.A., Yancy WS Jr, Weidenbacher H.J. (2016). Bariatric surgery and long-term durability of weight loss. JAMA Surg.

[9] Singh G., Krauthamer M., Bjalme-Evans M. (2022). Wegovy (semaglutide): a new weight loss drug for chronic weight management. J Invest Med.

[10] Moiz A., Levett J.Y., Filion K.B., Peri K., Reynier P., Eisenberg M.J. (2024). Long-term efficacy and safety of once-weekly semaglutide for weight loss in patients without diabetes: a systematic review and meta-analysis of randomized controlled trials. Am J Cardiol.

[11] Wharton S., Calanna S., Davies M., Dicker D., Goldman B., Lingvay I. (2022). Gastrointestinal tolerability of once-weekly semaglutide 2.4 mg in adults with overweight or obesity, and the relationship between gastrointestinal adverse events and weight loss. Diabetes Obes Metabol.

[12] Hasselbalch R.B., Andrea M.K., Nolsoe C.V., Hindborg M., Yazdanfard P.D.W., Sorensen K.K. (2025). Association between socioeconomic factors and semaglutide use for weight loss: a population-based cross-sectional study in Denmark. Lancet Reg Health Eur.

[13] Friedrichsen M., Breitschaft A., Tadayon S., Wizert A., Skovgaard D. (2021). The effect of semaglutide 2.4 mg once weekly on energy intake, appetite, control of eating, and gastric emptying in adults with obesity. Diabetes Obes Metabol.

[14] O'Neil P.M., Birkenfeld A.L., McGowan B., Mosenzon O., Pedersen S.D., Wharton S. (2018). Efficacy and safety of semaglutide compared with liraglutide and placebo for weight loss in patients with obesity: a randomised, double-blind, placebo and active controlled, dose-ranging, phase 2 trial. Lancet.

[15] Wilding J.P.H., Batterham R.L., Calanna S., Davies M., Van Gaal L.F., Lingvay I. (2021). Once-weekly semaglutide in adults with overweight or obesity. N Engl J Med.

[16] Davies M., Faerch L., Jeppesen O.K., Pakseresht A., Pedersen S.D., Perreault L. (2021). Semaglutide 2.4 mg once a week in adults with overweight or obesity, and type 2 diabetes (STEP 2): a randomised, double-blind, double-dummy, placebo-controlled, phase 3 trial. Lancet.

[17] Pi-Sunyer X., Astrup A., Fujioka K., Greenway F., Halpern A., Krempf M. (2015). A randomized, controlled trial of 3.0 mg of liraglutide in weight management. N Engl J Med.

[18] Torgerson J.S., Hauptman J., Boldrin M.N., Sjostrom L. (2004). XENical in the prevention of diabetes in obese subjects (XENDOS) study: a randomized study of orlistat as an adjunct to lifestyle changes for the prevention of type 2 diabetes in obese patients. Diabetes Care.

[19] Gadde K.M., Allison D.B., Ryan D.H., Peterson C.A., Troupin B., Schwiers M.L. (2011). Effects of low-dose, controlled-release, phentermine plus topiramate combination on weight and associated comorbidities in overweight and obese adults (CONQUER): a randomised, placebo-controlled, phase 3 trial. Lancet.

[20] Greenway F.L., Fujioka K., Plodkowski R.A., Mudaliar S., Guttadauria M., Erickson J. (2010). Effect of naltrexone plus bupropion on weight loss in overweight and obese adults (COR-I): a multicentre, randomised, double-blind, placebo-controlled, phase 3 trial. Lancet.

[21] Wing R.R., Lang W., Wadden T.A., Safford M., Knowler W.C., Bertoni A.G. (2011). Benefits of modest weight loss in improving cardiovascular risk factors in overweight and obese individuals with type 2 diabetes. Diabetes Care.

[22] Pradeepa R., Anjana R.M., Joshi S.R., Bhansali A., Deepa M., Joshi P.P. (2015). Prevalence of generalized & abdominal obesity in urban & rural India--the ICMR-INDIAB Study (Phase-I) [ICMR-INDIAB-3]. Indian J Med Res.

[23] Unnikrishnan R., Anjana R.M., Mohan V. (2014). Diabetes in south Asians: is the phenotype different?. Diabetes.

[24] Yajnik C.S., Yudkin J.S. (2004). The Y-Y paradox. Lancet.

