# Supplementary material for: Efficacy and safety of semaglutide injection in comparison with reference semaglutide for chronic weight management in indian adults with obesity: A phase III randomized non-inferiority trial
Source: Metabol Open. 2026 Mar 23;30:100460. doi: 10.1016/j.metop.2026.100460 (PMC13054606; doi:10.1016/j.metop.2026.100460)
Supplement: Multimedia component 1 [file mmc1.docx]

**CONSORT 2010 Checklist of Information to Include When Reporting a Randomised Trial**

*Efficacy and Safety of Semaglutide Injection in Comparison with Reference Semaglutide
for Weight Management in Indian Adults with Obesity: A Phase III Randomized Non-Inferiority Trial*

| **Section/Topic** | **Item No** | **Checklist Item** | **Reported on Page/Section** |
| --- | --- | --- | --- |
| **Title and Abstract** | | | |
|  | 1a | Identification as a randomised trial in the title | Title: Page 1 |
|  | 1b | Structured summary of trial design, methods, results, and conclusions | Abstract: Page 1 |
| **Introduction** | | | |
| *Background and objectives* | 2a | Scientific background and explanation of rationale | Introduction: Page 1-2 |
|  | 2b | Specific objectives or hypotheses | Introduction: Page 2 |
| **Methods** | | | |
| *Trial design* | 3a | Description of trial design (such as parallel, factorial) including allocation ratio | Methods- Study Design: Page 3 |
|  | 3b | Important changes to methods after trial commencement, with reasons | Not applicable — no changes reported |
| *Participants* | 4a | Eligibility criteria for participants | Methods- Participants: Page 3 |
|  | 4b | Settings and locations where data were collected | Methods- Study Design: Page 3 |
| *Interventions* | 5 | The interventions for each group with sufficient details to allow replication, including how and when they were actually administered | Methods- Procedure: Page 4 |
| *Outcomes* | 6a | Completely defined pre-specified primary and secondary outcome measures, including how and when they were assessed | Results- Page 5-12 |
|  | 6b | Any changes to trial outcomes after the trial commenced, with reasons | Not reported — no changes indicated |
| *Sample size* | 7a | How sample size was determined | Methods- Statistical analysis: Page 3 |
|  | 7b | When applicable, explanation of any interim analyses and stopping guidelines | Not applicable — no interim analyses |
| **Randomisation** | | | |
| *Sequence generation* | 8a | Method used to generate the random allocation sequence | Methods- Randomization and Masking: Page 4 |
|  | 8b | Type of randomisation; details of any restriction (such as blocking and block size) | Methods- Randomization and Masking: Page 4 |
| *Allocation concealment mechanism* | 9 | Mechanism used to implement the random allocation sequence, describing any steps taken to conceal the sequence until interventions were assigned | Methods- Randomization and Masking: Page 4 |
| *Implementation* | 10 | Who generated the random allocation sequence, who enrolled participants, and who assigned participants to interventions | Methods- Randomization and Masking: Page 4 |
| *Blinding* | 11a | If done, who was blinded after assignment to interventions (e.g., participants, care providers, those assessing outcomes) and how | Methods- Study Design: Open-label design — no blinding performed: Page 3 |
|  | 11b | If relevant, description of the similarity of interventions | Methods- Procedure: Page 4 |
| *Statistical methods* | 12a | Statistical methods used to compare groups for primary and secondary outcomes | Methods- Statistical analysis: Page 3 |
|  | 12b | Methods for additional analyses, such as subgroup analyses and adjusted analyses | Methods- Statistical analysis: Page 3 |
| **Results** | | | |
| *Participant flow* | 13a | For each group, the numbers of participants who were randomly assigned, received intended treatment, and were analysed for the primary outcome | Results- Page 5 |
|  | 13b | For each group, losses and exclusions after randomisation, together with reasons | Results- Page 5 |
|  |  | CONSORT flow diagram | Results- Figure 1: Page 5 |
| *Recruitment* | 14a | Dates of recruitment and follow-up | Results- Page 5 |
|  | 14b | Why the trial ended or was stopped | NA |
| *Baseline data* | 15 | A table showing baseline demographic and clinical characteristics for each group | Results- Table 1: Page 6 |
| *Numbers analysed* | 16 | For each group, number of participants (denominator) included in each analysis and whether the analysis was by original assigned groups | Results- Page 5 |
| *Outcomes and estimation* | 17a | For each primary and secondary outcome, results for each group, and the estimated effect size and its precision (such as 95% confidence interval) | Results- Page 5-12 |
|  | 17b | For binary outcomes, presentation of both absolute and relative effect sizes is recommended | Absolute measures presented |
| *Ancillary analyses* | 18 | Results of any other analyses performed, including subgroup analyses and adjusted analyses, distinguishing pre-specified from exploratory | Results- Page 8: Table 3 (All vs diabetics) |
| *Harms* | 19 | All important harms or unintended effects in each group | Results- Page 9 |
| **Discussion** | | | |
| *Limitations* | 20 | Trial limitations, addressing sources of potential bias, imprecision, and, if relevant, multiplicity of analyses | Discussion- Page 12-14 |
| *Generalisability* | 21 | Generalisability (external validity, applicability) of the trial findings | Discussion- Page 12-14 |
| *Interpretation* | 22 | Interpretation consistent with results, balancing benefits and harms, and considering other relevant evidence | Discussion- Page 12-14 |
| **Other information** | | | |
| *Registration* | 23 | Registration number and name of trial registry | Methods- Ethical considerations: Page 3 |
| *Protocol* | 24 | Where the full trial protocol can be accessed, if available | Methods- Ethical considerations: Page 3 |
| *Funding* | 25 | Sources of funding and other support (such as supply of drugs), role of funders | Funding- Page 14 |
