# Supplementary material for: Efficacy and safety of semaglutide injection in comparison with reference semaglutide for chronic weight management in indian adults with obesity: A phase III randomized non-inferiority trial
Source: Metabol Open. 2026 Mar 23;30:100460. doi: 10.1016/j.metop.2026.100460 (PMC13054606; doi:10.1016/j.metop.2026.100460)
Supplement: Multimedia component 2 [file mmc2.docx]

**Supplementary Material**

**Supplementary Table S1.** Primary and Secondary Efficacy Outcomes (Per-Protocol Population)

| **Outcome** | **Test Product (N=171)** | **Reference (N=85)** | **LSM Difference (95% CI)** | **P-value** |
| --- | --- | --- | --- | --- |
| **Primary Endpoint** | | | | |
| % weight change, Week 24, LSM (SE) | −11.78 (0.29) | −12.11 (0.41) | 0.33 (−0.66, 1.32) | 0.513 |
| **Secondary Endpoints: % Weight Change Over Time, LSM (SE)** | | | | |
| Week 4 | −1.92 (0.30) | −1.80 (0.44) | −0.12 (−1.17, 0.93) | 0.826 |
| Week 8 | −3.44 (0.29) | −3.30 (0.41) | −0.14 (−1.13, 0.85) | 0.782 |
| Week 12 | −5.62 (0.29) | −5.34 (0.42) | −0.29 (−1.28, 0.71) | 0.572 |
| Week 16 | −7.66 (0.29) | −7.40 (0.41) | −0.25 (−1.24, 0.74) | 0.615 |
| Week 20 | −10.23 (0.29) | −9.96 (0.41) | −0.26 (−1.26, 0.73) | 0.600 |
| **Responder Analysis at Week 24, n (%)** | | | | |
| ≥5% weight loss | 163 (95.3%) | 80 (94.1%) | — | 0.623 |
| ≥10% weight loss | 130 (76.0%) | 66 (77.6%) | — | 0.768 |

*Abbreviations: CI, confidence interval; LSM, least squares mean; PP, per-protocol; SE, standard error. The PP population included participants who completed the study without major protocol deviations. Results are consistent with the primary mITT analysis, confirming non-inferiority.*

**Supplementary Table S2.** Anthropometric and Metabolic Parameters (Per-Protocol Population)

| **Parameter** | **Test Product (N=171)** | **Reference (N=85)** | **Difference (95% CI)** | **P-value** |
| --- | --- | --- | --- | --- |
| **All Patients** | | | | |
| BMI change, kg/m², LSM (SE) | −3.98 (0.10) | −4.08 (0.15) | 0.11 (−0.24, 0.46) | 0.548 |
| Waist circumference change, cm, LSM (SE) | −10.92 (0.41) | −10.54 (0.58) | −0.38 (−1.77, 1.01) | 0.591 |
| **Patients with Type 2 Diabetes (PP: n=72)** | | | | |
| HbA1c change, %, LSM (SE) | −1.64 (0.13) | −1.83 (0.17) | 0.19 (−0.24, 0.62) | 0.384 |
| FPG change, mg/dL, LSM (SE) | −43.82 (4.68) | −51.24 (6.45) | 7.42 (−8.52, 23.36) | 0.358 |
| 2-hour PPG change, mg/dL, LSM (SE) | −64.92 (6.70) | −71.10 (9.19) | 6.17 (−16.19, 28.54) | 0.588 |

*Abbreviations: BMI, body mass index; CI, confidence interval; FPG, fasting plasma glucose; HbA1c, glycated hemoglobin; LSM, least squares mean; PP, per-protocol; PPG, postprandial glucose; SE, standard error.*

**Supplementary Table S3.** Immunogenicity Assessment

| **Parameter** | **Test Product (N=188)** | **Reference Semaglutide (N=94)** | **Total (N=282)** |
| --- | --- | --- | --- |
| **Anti-drug antibodies (ADA)** | | | |
| Baseline (pre-dose) positive, n (%) | 3 (1.60%) | 0 (0%) | 3 (1.06%) |
| Week 24 (post-dose) positive, n (%) | 0 (0%) | 1 (1.06%) | 1 (0.35%) |
| Total ADA positive (either timepoint), n (%) | 3 (1.60%) | 1 (1.06%) | 4 (1.42%) |
| **Neutralizing antibodies (NAb)*** | | | |
| Baseline (pre-dose) positive, n (%) | 2 (1.06%) | 0 (0%) | 2 (0.71%) |
| Week 24 (post-dose) positive, n (%) | 0 (0%) | 0 (0%) | 0 (0%) |
| **Treatment-induced responses** | | | |
| Treatment-induced ADA, n (%) | 0 (0%) | 0 (0%) | 0 (0%) |
| Treatment-induced NAb, n (%) | 0 (0%) | 0 (0%) | 0 (0%) |

*Abbreviations: ADA, anti-drug antibodies; NAb, neutralizing antibodies. Blood samples were collected at baseline (Day 0) and Week 24 for immunogenicity assessment.*

**Supplementary Table S4. Dose-Escalation Schedule and Patient Numbers by Visit and Treatment Group**

| **Study Period** | **Dose (mg/week)** | **Visit** | **Test Product (N at visit)** | **Reference Semaglutide (N at visit)** |
| --- | --- | --- | --- | --- |
| Week 0–4 | 0.25 | Visit 2 (Day 0) | 188 | 94 |
| Week 4–8 | 0.5 | Visit 3 (Week 4) | 180 | 89 |
| Week 8–12 | 1.0 | Visit 4 (Week 8) | 179 | 87 |
| Week 12–16 | 1.7 | Visit 5 (Week 12) | 178 | 87 |
| Week 16–20 | 2.4 | Visit 6 (Week 16) | 177 | 87 |
| Week 20–24 (Maintenance) | 2.4 | Visit 7 (Week 20) Visit 8 (Week 24) | 177 | 87 |

*All participants who completed the study achieved the target maintenance dose of 2.4 mg once weekly. No dose reductions were required in either group.*

**Supplementary Table S5. Adverse Events by System Organ Class and Preferred Term (Safety Population)**

| **SOC / Preferred Term** | **Test Product N (%)** | **Test Events** | **Reference Semaglutide N (%)** | **Ref Events** |
| --- | --- | --- | --- | --- |
| **Gastrointestinal disorders** |  |  |  |  |
| Diarrhoea | 45 (23.94%) | 70 | 18 (19.15%) | 20 |
| Vomiting | 38 (20.21%) | 64 | 15 (15.96%) | 21 |
| Nausea | 21 (11.17%) | 47 | 14 (14.89%) | 30 |
| Bloating | 10 (5.32%) | 25 | 05 (5.32%) | 14 |
| Acidity | 14 (7.45%) | 17 | 05 (5.32%) | 07 |
| Dyspepsia | 05 (2.66%) | 10 | 04 (4.26%) | 05 |
| Constipation | 06 (3.19%) | 07 | 05 (5.32%) | 14 |
| Abdominal pain | 05 (2.66%) | 06 | 02 (2.13%) | 02 |
| Gastritis | 02 (1.06%) | 02 | 03 (3.19%) | 03 |
| Abdominal distension | 02 (1.06%) | 02 | 01 (1.06%) | 01 |
| Epigastric discomfort | 01 (0.53%) | 01 | 00 (0.00%) | 00 |
| Flatulence | 02 (1.06%) | 02 | 00 (0.00%) | 00 |
| Heartburn | 01 (0.53%) | 01 | 00 (0.00%) | 00 |
| Toothache | 01 (0.53%) | 01 | 00 (0.00%) | 00 |
| **General disorders and administration site conditions** |  |  |  |  |
| Pyrexia | 19 (10.11%) | 19 | 03 (3.19%) | 03 |
| Asthenia | 10 (5.32%) | 14 | 03 (3.19%) | 04 |
| Fatigue | 02 (1.06%) | 02 | 00 (0.00%) | 00 |
| Injection site pain | 00 (0.00%) | 00 | 01 (1.06%) | 01 |
| **Nervous system disorders** |  |  |  |  |
| Headache | 08 (4.26%) | 08 | 05 (5.32%) | 05 |
| Somnolence | 02 (1.06%) | 02 | 00 (0.00%) | 00 |
| Dizziness | 00 (0.00%) | 00 | 02 (2.13%) | 02 |
| Ageusia | 00 (0.00%) | 00 | 01 (1.06%) | 01 |
| **Metabolism and nutrition disorders** |  |  |  |  |
| Decreased appetite | 08 (4.26%) | 08 | 02 (2.13%) | 02 |
| **Musculoskeletal and connective tissue disorders** |  |  |  |  |
| Body pain | 04 (2.13%) | 04 | 01 (1.06%) | 01 |
| **Skin and subcutaneous tissue disorders** |  |  |  |  |
| Alopecia | 03 (1.60%) | 03 | 00 (0.00%) | 00 |
| Pruritus | 00 (0.00%) | 00 | 01 (1.06%) | 01 |
| Skin allergy | 00 (0.00%) | 00 | 01 (1.06%) | 01 |
| **Infections and infestations** |  |  |  |  |
| Nasopharyngitis | 01 (0.53%) | 01 | 02 (2.13%) | 02 |
| **Respiratory, thoracic and mediastinal disorders** |  |  |  |  |
| Cough | 01 (0.53%) | 01 | 00 (0.00%) | 00 |

*Percentages based on safety population in respective treatment group. Adverse events coded using MedDRA. All AEs resolved without sequelae.*

***Supplementary File:*** CONSORT Checklist
